# Identification of a Novel C-Terminal Truncated WT1 Isoform with Antagonistic Effects against Major WT1 Isoforms

**DOI:** 10.1371/journal.pone.0130578

**Published:** 2015-06-19

**Authors:** Naoya Tatsumi, Nozomi Hojo, Hiroyuki Sakamoto, Rena Inaba, Nahoko Moriguchi, Keiko Matsuno, Mari Fukuda, Akihide Matsumura, Seiji Hayashi, Soyoko Morimoto, Jun Nakata, Fumihiro Fujiki, Sumiyuki Nishida, Hiroko Nakajima, Akihiro Tsuboi, Yoshihiro Oka, Naoki Hosen, Haruo Sugiyama, Yusuke Oji

**Affiliations:** 1 Department of Functional Diagnostic Science, Osaka University Graduate School of Medicine, Osaka, Japan; 2 Department of Surgery, National Hospital Organization, Kinki-Chuo Chest Medical Center, Osaka, Japan; 3 National Hospital Organization, Kinki-Chuo Chest Medical Center, Osaka, Japan; 4 Department of Cancer Immunology, Osaka University Graduate School of Medicine, Osaka, Japan; 5 Department of Cancer Immunotherapy, Osaka University Graduate School of Medicine, Osaka, Japan; 6 Department of Respiratory Medicine, Allergy and Rheumatic Disease, Osaka University Graduate School of Medicine, Osaka, Japan; 7 Department of Cancer Stem Cell Biology, Osaka University Graduate School of Medicine, Osaka, Japan; University of Bristol, UNITED KINGDOM

## Abstract

The Wilms’ tumor gene *WT1* consists of 10 exons and encodes a zinc finger transcription factor. There are four major WT1 isoforms resulting from alternative splicing at two sites, exon 5 (17AA) and exon 9 (KTS). All major WT1 isoforms are overexpressed in leukemia and solid tumors and play oncogenic roles such as inhibition of apoptosis, and promotion of cell proliferation, migration and invasion. In the present study, a novel alternatively spliced WT1 isoform that had an extended exon 4 (designated as exon 4a) with an additional 153 bp (designated as 4a sequence) at the 3’ end was identified and designated as an Ex4a(+)WT1 isoform. The insertion of exon 4a resulted in the introduction of premature translational stop codons in the reading frame in exon 4a and production of C-terminal truncated WT1 proteins lacking zinc finger DNA-binding domain. Overexpression of the truncated Ex4a(+)WT1 isoform inhibited the major WT1-mediated transcriptional activation of anti-apoptotic *Bcl-xL* gene promoter and induced mitochondrial damage and apoptosis. Conversely, suppression of the Ex4a(+)WT1 isoform by Ex4a-specific siRNA attenuated apoptosis. These results indicated that the Ex4a(+)WT1 isoform exerted dominant negative effects on anti-apoptotic function of major WT1 isoforms. Ex4a(+)WT1 isoform was endogenously expressed as a minor isoform in myeloid leukemia and solid tumor cells and increased regardless of decrease in major WT1 isoforms during apoptosis, suggesting the dominant negative effects on anti-apoptotic function of major WT1 isoforms. These results indicated that Ex4a(+)WT1 isoform had an important physiological function that regulated oncogenic function of major WT1 isoforms.

## Introduction

The Wilms’ tumor gene *WT1* was originally isolated as a tumor suppressor gene in Wilms’ tumor, a childhood kidney cancer [1, 2]. However, it was reported that the wild-type *WT1* gene is overexpressed in leukemia and various kinds of solid cancers including lung [3], colon [4] and pancreatic cancers [5]. Furthermore, it was proposed that the wild-type WT1 plays oncogenic rather than tumor-suppressor functions in leukemogenesis and tumorigenesis [6].

The *WT1* gene consists of 10 exons and encodes a zinc finger transcription factor. The N-terminal region of WT1 protein contains a proline and glutamine rich domain involved in transcriptional regulation, self-association, and RNA recognition [7–9], and the C-terminal region of WT1 protein contains four zinc fingers that are encoded by exons 7 to 10 and that bind to DNA and RNA [10]. The zinc finger domain of WT1 can bind to GC-rich sequences, such as the EGR-1 consensus sequence (5’-GCG(T/G)GGGCG-3’) [11], the WTE motif (5'-GCGTGGGAGT-3') [12], or (TCC)n motif [13]. Many genes responsible for cell growth and apoptosis such as *Bcl-2*, *Bcl-xL*, *BFL1*, and *c-myc* have been identified as downstream targets of WT1 [14–17].

The *WT1* transcript contains two alternative splicing regions corresponding to the cassette exon 5 (17AA) and the three last codons of exon 9 (KTS), resulting in the production of four major WT1 protein isoforms [17AA(+)KTS(+), 17AA(+)KTS(-), 17AA(-)KTS(+), and 17AA(-)KTS(-)] [18]. Unlike the KTS, the exon 5 (17AA) is only present in mammals [19, 20]. However, the mammal-specific 17AA is not required for any of mammal-specific processes such as embryonic implantation or lactation and mice lacking 17AA normally develop and fertile [21]. It has been shown that all major *WT1* isoforms are overexpressed in leukemia and solid tumors, where different major isoforms have different oncogenic functions. 17AA(+)WT1 isoforms [17AA(+)KTS(+) and 17AA(+)KTS(-)] exert their anti-apoptotic function through stabilization of mitochondrial membrane in leukemia [22] and solid tumors [23]. 17AA(-)KTS(-)WT1 isoform induces cytoskeletal changes and promotes cell migration and invasion [24].

The *WT1* transcript also contains three alternative translational start codons, the regular AUG codon, an upstream in-frame CUG codon that produces larger WT1 proteins [25], and a downstream in-frame AUG codon that produces smaller WT1 proteins [26]. The larger WT1 protein translated from upstream CUG codon is not essential for normal development and reproduction in mice [27]. In addition, transcriptional initiation from an alternative promoter located in intron 1 results in the production of a smaller N-terminal truncated WT1 protein (AWT1 also known as sWT1) [28–29] and the N-terminal truncated WT1 has more oncogenic potential than wild-type WT1 in leukemia cells [28]. An additional WT1 isoform generated by alternative transcriptional initiation at the end of intron 5 was identified in human cancer cells and the shorter transcript encodes N-terminal truncated protein lacking exons 1 to 5 [30]. In total, at least 36 different isoforms of WT1 protein are theoretically produced by combinations of alternative transcriptional initiation, alternative splicing, RNA editing [31], and alternative translational initiation.

Here we report the identification of a novel alternatively spliced isoform of WT1, designated as an Ex4a(+)WT1 isoform. The Ex4a(+)WT1 transcript contains the extended exon 4 with an additional 153 bp at the 3’ end, resulting in the introduction of in-frame premature translational stop codons in the reading frame in exon 4a and production of at least two C-terminal truncated proteins lacking entire zinc finger DNA-binding domain. Furthermore, we show that the truncated Ex4a(+)WT1 exerts dominant negative effects on anti-apoptotic function of major WT1 isoforms during apoptosis and has a physiological function to promote apoptosis.

## Results

### Identification of a novel splicing isoform of WT1

**Fig 1:**
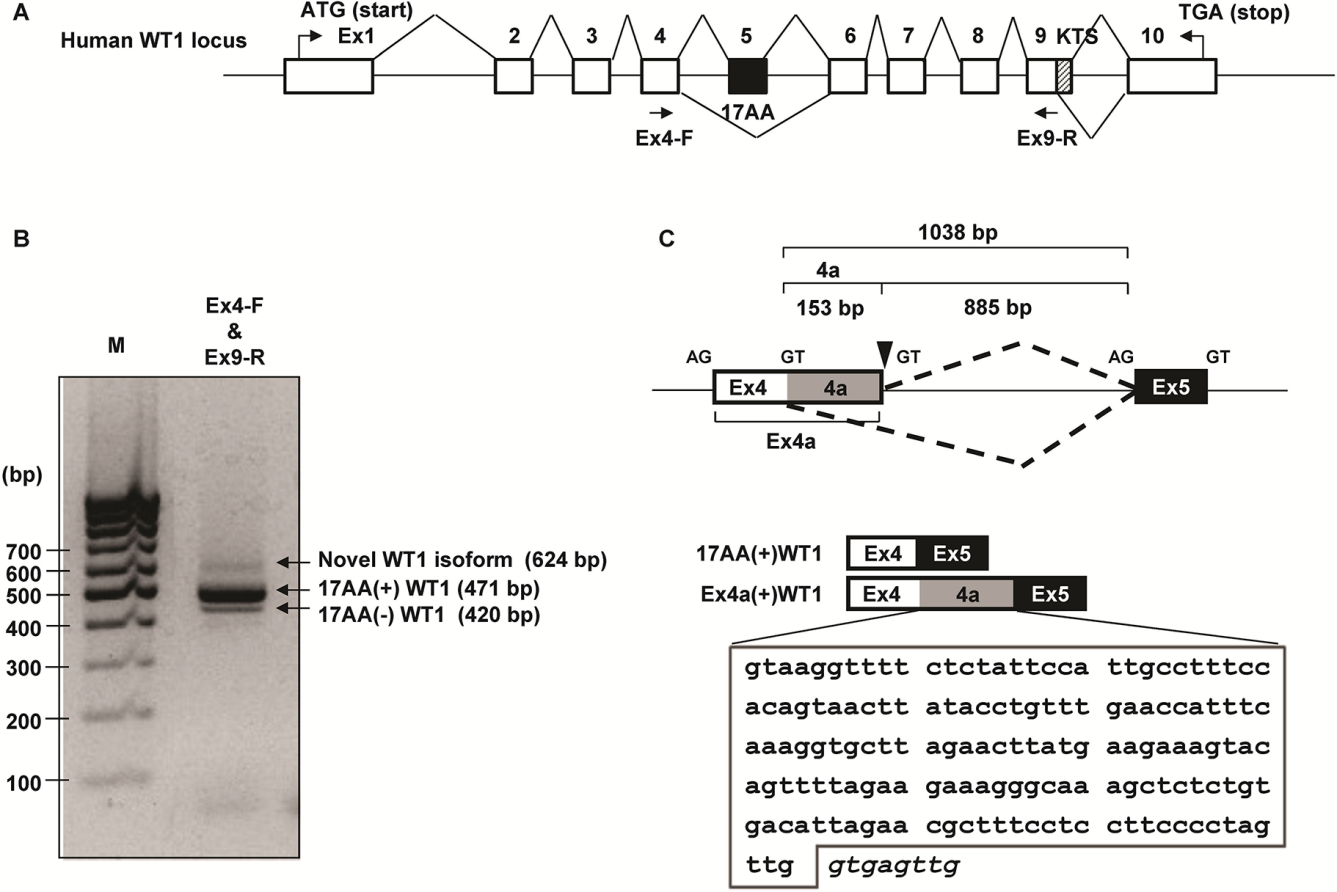
Isolation of a novel WT1 isoform. (**A**) Schematic representation of the human WT1 genomic structure (not to scale). Primers used for PCR amplification are indicated with arrows. Exons are shown as open boxes. Alternative splicing exon 5 (17AA) and KTS region in exon 9 are represented as black and hatched boxes, respectively. Translational start (ATG) and stop codons (TGA) are indicated. (**B**) Agarose gel electrophoresis of WT1 PCR products amplified using the primers indicated in (A) is shown. Bands were visualized by Gel-red staining. A novel transcript of 624 bp together with major WT1 isoforms, 17AA(+)WT1 (471 bp) and 17AA(-)WT1 (420 bp) were detected. Lane M, molecular marker (100 bp DNA ladder). (**C**) Upper, schematic representation of the WT1 genomic region encompassing exons 4 to 5, including the alternative splicing site in exon 4. Lower, schematic representation of the 17AA(+) and Ex4a(+)WT1 isoforms. Alternatively spliced exons 4a and 5 are shown in shaded and black boxes, respectively. DNA sequence of the newly identified 4a of 153 bp is expanded in the lower and surrounded by a solid line. Genomic sequences that follow immediately after the end of the 4a are shown in italic.

cDNA from K562 cells was amplified using WT1-specific primer pair located in exons 4 and 9 of the WT1 cDNA (Genbank accession No. NG_009272) (**Fig 1A**). A novel transcript of 624 bp was amplified together with major WT1 isoforms, 17AA(+)WT1 (471 bp) and 17AA(-)WT1 (420 bp) (**Fig 1B**). Sequence analysis showed that the novel transcript included the extended exon 4 (designated as Ex4a) with additional 153 bp (designated as 4a sequence) at the 3’ end (**Fig 1C**). Splice donor consensus sequences GT was detected precisely adjacently to the 3’ end of Ex4a (**Fig 1C**). WT1 genomic sequence spanning exons 4 to 5 was analyzed for 5’ and 3’ splice sites using Alternative Splice Site Predictor Database (ASSP, http://wangcomputing.com/assp/[32]). The database analysis predicted the novel splice site in the intron 4 as an alternative 5’ splice donor site. These results indicated that the novel WT1 transcript was a new isoform of WT1 with additional 4a of 153 bp that was generated by new alternative splicing. It was therefore designated as Ex4a(+)WT1 isoform.

### Cloning and sequence analysis of a full-length Ex4a(+)WT1 isoform

**Fig 2:**
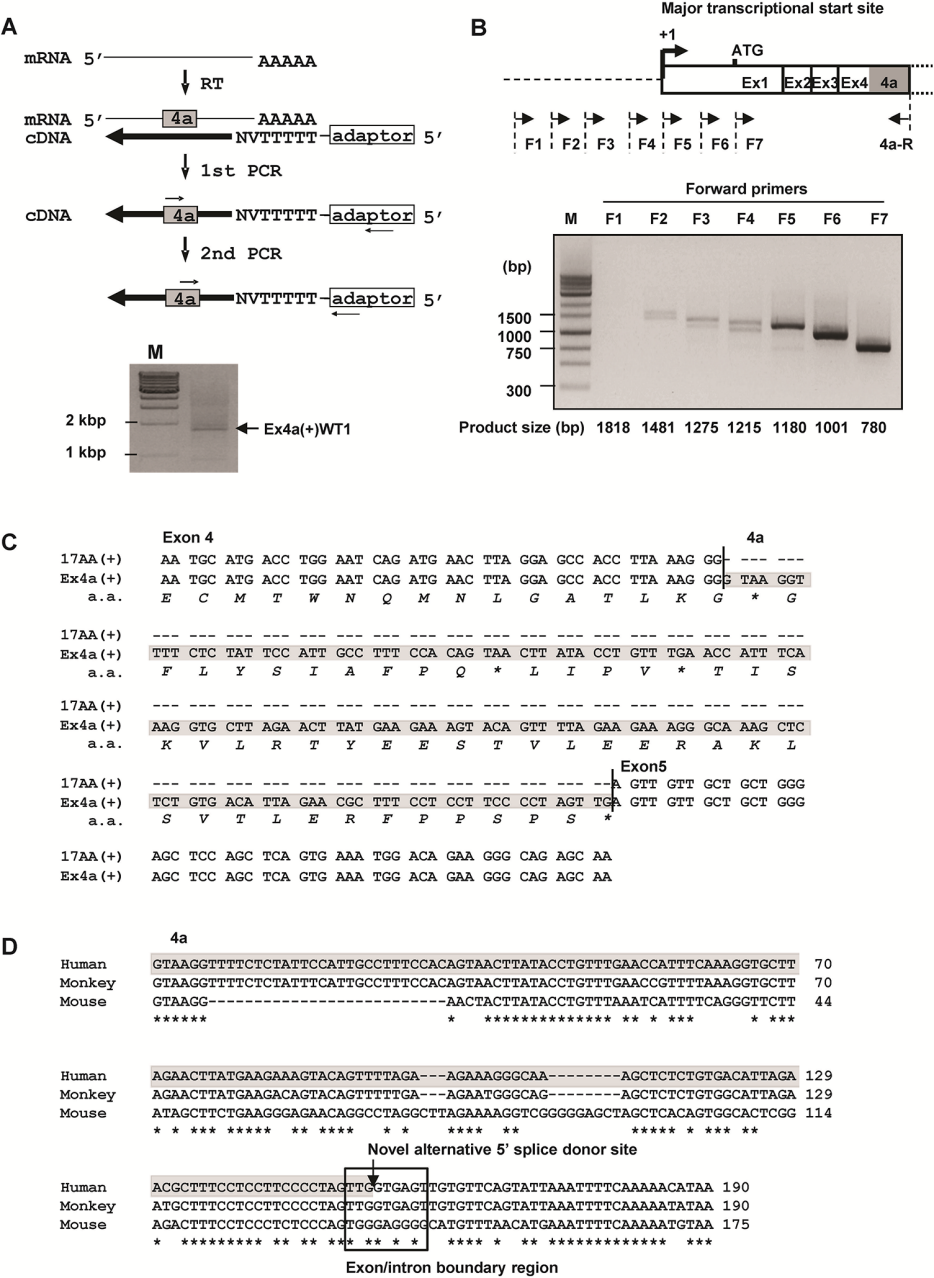
Cloning and sequence analysis of full-length Ex4a(+) WT1 isoform. (**A**) 3’ rapid amplification of cDNA ends (3’ RACE) assay. Upper, 3’ RACE overview. cDNA was synthesized by using a 3’ RACE adaptor-dT primer. First PCR was performed by using exon 4a-specific forward and adaptor outer reverse primers. Second nested PCR was performed by using the first PCR product as a template with the nested Ex4a(+)WT1 cDNA-specific forward and the nested adaptor inner reverse primers. Lower, Agarose gel electrophoresis of the second nested PCR products is shown. Lane M, molecular marker (1 kbp DNA ladder). (**B**) Transcriptional start site of Ex4a(+)WT1. Upper, Schematic representation of the primer locations used to determine the transcriptional start site of Ex4a(+)WT1. Four (F1, F2, F3 and F4) and three (F5, F6, and F7) forward primers are located upstream and downstream, respectively, of the major transcriptional start site of WT1. Reverse primer (4a-R) is located in Exon 4a. The arrows indicate the primer positions used for PCR. +1 represents the major transcription start site of WT1. ATG indicates translational start codon. Lower, Agarose gel electrophoresis of PCR products amplified by using one each of 7 forward and 4a-R primers is shown. Lane M, molecular marker (1 kbp DNA ladder). (**C**) Alignment of nucleotide sequence of human 17AA(+)WT1 (upper lane) and Ex4a(+)WT1 (lower lane), and amino-acid (aa) sequences of exons 4 and 4a are shown. Asterisk indicates translational stop codon. Gaps are represented by dashes. The 4a sequence is marked in shaded grey. (**D**) Alignment of nucleotide sequence of the 4a and a part of intron 4 of WT1 in human, monkey, and mouse. The alignment is generated by CLUSTAL2.1 database with default parameters. The asterisks represent nucleotides identical to human and dashes represent an alignment gap. The 4a sequence of human WT1 is marked in shaded grey. Arrow indicates a novel alternative 5’ splice donor site. Genomic sequences of the exon 4a/intron 4 boundary region are highlighted in box.

To determine the 3’ exon structure of the Ex4a(+)WT1 isoform, 3’ RACE analysis was performed (**Fig 2A**). Total RNA from K562 cells was reverse transcribed with adaptor-dT primer, and partial WT1 cDNA was amplified using WT1 4a-specific forward and adaptor-specific reverse primers. The PCR products were cloned and sequenced. The sequences of 3’ exon of the Ex4a(+)WT1 isoform was identical to those of 17AA(+)KTS(+)WT1 isoform with an additional 4a sequence.

Next, to confirm the transcription start site of the Ex4a(+)WT1 isoform, seven different forward primers were designed upstream (F1, F2, F3 and F4) and downstream (F5, F6 and F7) of the major transcription start site of WT1 that was registered in Database of Transcriptional Start Sites (DBTSS, http://dbtss.hgc.jp/, [33]), and PCR was performed by using one each of 7 forward primers and exon 4a-specific reverse (4a-R) primer (**Fig 2B**). Clear PCR bands were detected by using F5, F6, and F7 primers, whereas no or faint PCR bands were detected by using F1, F2, F3, and F4 primers (**Fig 2B**). These results indicated that the transcription of the Ex4a(+)WT1 isoform was predominantly initiated from the major transcription start site of WT1. Furthermore, to determine the 5’ exon sequence of the Ex4a(+)WT1 isoform, the PCR products amplified by using F5 and 4a-R were cloned and sequenced (**Fig 2C**). The Ex4a(+)WT1 had the same 5’ exon sequence as that of 17AA(+)KTS(+)WT1 isoform.

Taken together, the full-length Ex4a(+)WT1 isoform had the same exon sequence as that of 17AA(+)KTS(+)WT1 with an additional 4a sequence (Genbank accession No. AB971668).

### 4a sequence is highly conserved in human and monkey, but not in mouse

The 4a sequence of 153 bp was compared among human, monkey, and mouse. The 4a sequence of 153 bp was conserved with 94.1% identity between human and monkey, and with 68.8% identity between human and mouse (**Fig 2D**). As shown by the highlighted box in Fig 2D, the alternative 5’ splice donor sequences (5’-TTG/GTGAGT-3’) at the exon 4a/intron 4 boundary were perfectly conserved between human and monkey, whereas they were less conserved between human and mouse. These results suggested the Ex4a(+)WT1 isoform was present in monkey but absent in mouse.

### Ex4a(+)WT1 isoform produces C-terminal truncated WT1 proteins

**Fig 3:**
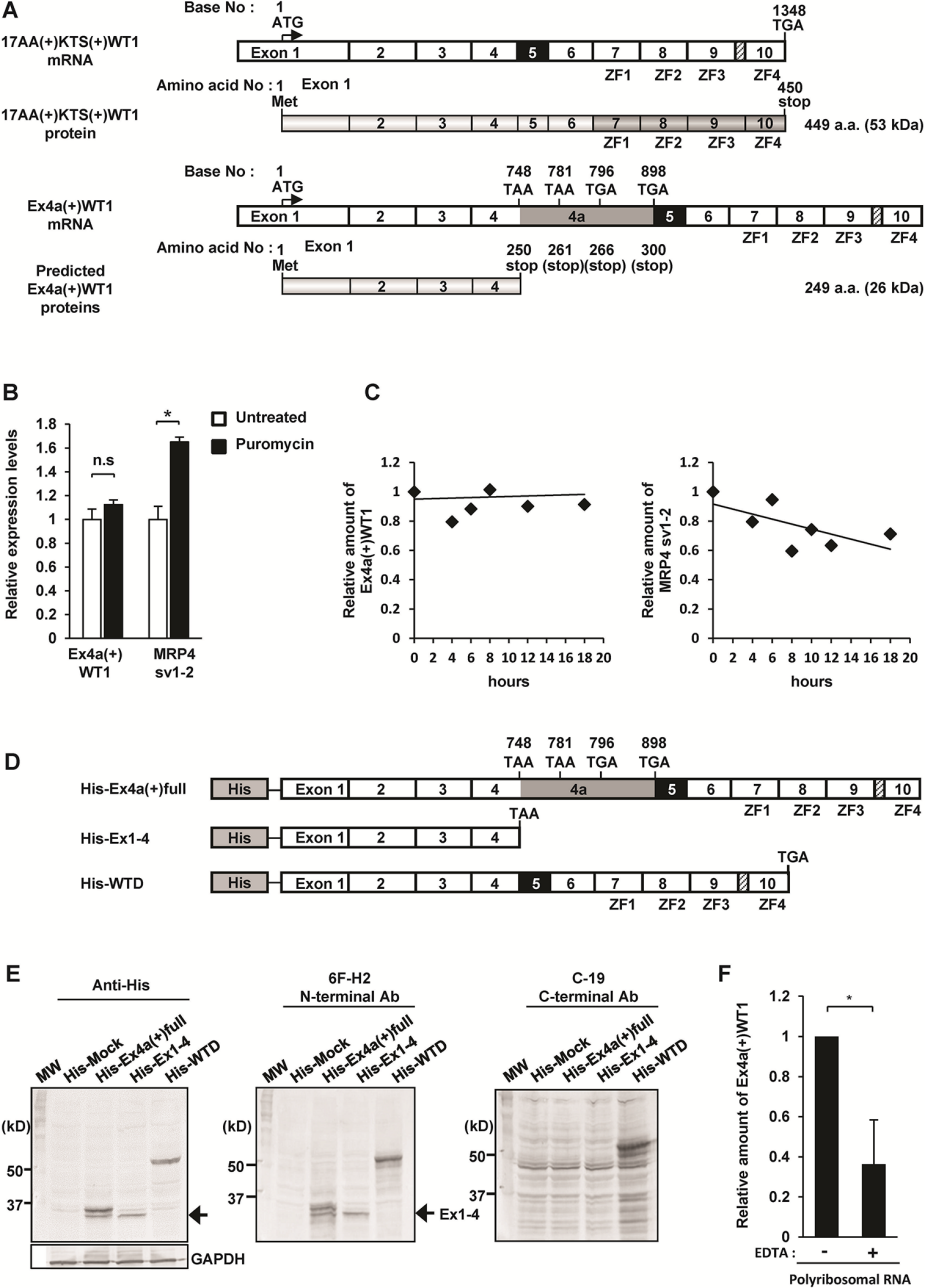
Ex4a(+)WT1 isoform produces C-terminal truncated WT1 proteins. (**A**) Schematic representation of 17AA(+)KTS(+)WT1 mRNA, structure of 17AA(+)KTS(+)WT1 protein, Ex4a(+)WT1 mRNA, and predicted structures of Ex4a(+)WT1 proteins. Translational start (ATG) and stop codons (TGA, TAA) are indicated. Four premature translational stop codons (two TAA and two TGA) are present in the reading frame in exon 4a. ZF represents zinc finger domain. (**B**) NMD inhibition by puromycin treatment. K562 cells were treated with 100 μg/ml of puromycin for 4 h to block NMD pathway and the amount of Ex4a(+)WT1 and MRP4 sv1-2 mRNA were determined by quantitative real-time PCR. The amount of each mRNA in puromycin-untreated K562 cells is defined as 1.0. Results are mean and S.D. of three independent experiments. *, p<0.05. (**C**) Stability of Ex4a(+)WT1 mRNA. K562 cells were treated with the transcription inhibitor ActD (5 μg/ml) and the amount of Ex4a(+)WT1 and MRP4 sv1-2 mRNA were determined by quantitative real-time PCR at indicated time points. The amount of Ex4a(+)WT1 and MRP4 sv1-2 mRNA were normalized to U6snRNA that were transcribed by RNA polymerase III and thus not blocked by ActD. The amount of each mRNA in ActD-untreated K562 cells (0 hours) is defined as 1.0. The trendlines are shown by black lines. (**D**) Schematic representation of three N-terminal His-tagged WT1 vectors. (**E**) Western blot analysis of total protein extracts from HT-1080 cells transfected with His-Mock, His-Ex4a(+)full, His-Ex1-4, or His-WTD expression vector. Transferred membrane was blotted with anti-His tag, 6F-H2 (specific for the N-terminal region of WT1 protein), or C-19 (specific for the C-terminal region of WT1 protein) antibody. MW represents molecular weight marker. Arrows indicate 30-KDa His-tagged truncated Ex1-4 WT1 protein. Results are representative of three independent experiments. (**F**) Endogenous Ex4a(+)WT1 mRNA is associated with polyribosomes. Polyribosomal fractions of K562 cells were purified by using the polyribosomal buffer with or without 100 mM EDTA (to release RNA from polyribosomes) and the amount of Ex4a(+)WT1 mRNA associated with polyribosomes was determined by quantitative real-time PCR using 4a-F and Ex6-R primer pair. The amount of Ex4a(+)WT1 mRNA in polyribosomes purified by using polyribosomal buffer without EDTA were defined as 1.0. Results are means and S.D. of three independent experiments. *, p<0.05.

The insertion of the 153 bp of 4a resulted in the introduction of 4 premature translation termination codons (PTCs) in Ex4a in the reading frame (**Fig 3A**). The predicted open reading frame of the Ex4a(+)WT1 mRNA was 747 bp in length and thus the transcript was predicted to encode a protein of 249 amino acids (molecular weight 26 kDa) that lacked C-terminus including zinc finger domain (**Fig 3A**).

There may be the possibility that transcripts containing PTC located more than 50–55 nt upstream of a last exon-exon junction, like Ex4a(+)WT1 transcript, are recognized and rapidly degraded by nonsense-mediated mRNA decay (NMD) pathway [34]. To examine whether or not the Ex4a(+)WT1 mRNA could be degraded by the NMD pathway, K562 cells were treated with puromycin for 4 h to block NMD pathway. Then, the amount of Ex4a(+)WT1 mRNA was determined by quantitative real-time PCR (**Fig 3B left**). The amount of two spliced isoforms of *MRP4* gene (MRP4 sv1-2), which has been shown to contain PTC and undergo degradation by NMD pathway in K562 cells [35], was also determined as a NMD-sensitive positive control gene (**Fig 3B right**). If the Ex4a(+)WT1 mRNA was recognized and rapidly degraded by NMD pathway, it would be expected that the amount of Ex4a(+)WT1 mRNA could be increased by puromycin treatment. However, the amount of Ex4a(+)WT1 mRNA remained unchanged by the block of NMD pathway, whereas the amount of the NMD-sensitive MRP4 sv1-2 mRNA was increased by puromycin treatment (**Fig 3B**). Furthermore, the stability of the Ex4a(+)WT1 mRNA in K562 cells following treatment with transcriptional inhibitor ActimomycinD (ActD) was examined (**Fig 3C**). The Ex4a(+)WT1 mRNA was stable for at least 18 h after inhibition of new transcription whereas the NMD-sensitive MRP4 sv1-2 mRNA decreased in time. These results indicated that the Ex4a(+)WT1 mRNA did not undergo degradation by NMD pathway.

To examine the truncated proteins consisting of the N-terminus of WT1 that was translated from the PTC-containing Ex4a(+)WT1 mRNA, one each of three kinds of N-terminal His-tagged vectors that expressed PTC-containing full-length Ex4a(+)17AA(+)KTS(+)WT1 (His-Ex4a(+)full), WT1 fragment from exons 1 to 4 (His-Ex1-4), or full-length 17AA(+)KTS(+)WT1 (His-WTD), or an empty vector (His-Mock) was transfected into HT-1080 cells and examined for protein expression (**Fig 3D and 3E**). His-Ex1-4, His-WTD, and His-Mock vectors were used as a control. His-Ex1-4-transfected cells produced approximately 30-kDa His-tagged protein (arrow) that was detected by anti-His tag and anti-WT1 (6F-H2) antibodies against the N-terminal region of WT1 protein, but not by anti-WT1 antibody (C-19) against the C-terminal region of WT1 protein. His-WTD-transfected cells produced 57-kDa His-WTD protein that was detected by anti-His tag, 6F-H2, and C-19 antibodies. On the other hand, His-Ex4a(+)full-transfected cells produced 30- and 36-kDa His-tagged proteins. The 30-kDa His-tagged protein corresponded to protein product from Ex1-4 transcript that was stopped at TAA stop codon at 748 bp, and 36-kDa His-tagged protein appeared to correspond to protein product of the transcript that was stopped at TGA stop codon at 898 bp. These results indicated that the full-length Ex4a(+)WT1 mRNA produced at least two truncated proteins consisting of exons 1 to 4, and exons 1 to 4a. The exact mechanisms by which translation does not stop at the first stop codon are unclear at present time. However, it might be due to the translational read-through of the first PTC [36].

These endogenous truncated WT1 proteins could not be detected by SDS-PAGE analysis of whole cell lysate from HT-1080 and K562 cells probably because of their low expression. To enrich low-abundance proteins, sub-cellular fractionation was performed (**Figure A in S1 Fig**) Nuclear and cytoplasmic fractions of K562 cells were isolated and WT1 protein expression was examined by SDS-PAGE. However, the endogenous truncated Ex4a(+)WT1 proteins could not be detected in any fractions. Next, WT1 protein expression was examined in Doxorubicin (Dox)-treated K562 cells, in which Ex4a(+)WT1 mRNA levels increased and major WT1 isoforms decreased. However, the endogenous truncated Ex4a(+)WT1 proteins could not be detected in Dox-treated cell lysate (**Figure B in S1 Fig**). These results suggested that endogenous Ex4a(+)WT1 proteins was too low to be detected by SDS-PAGE. Therefore, to confirm whether endogenous EX4a(+)WT1 mRNA was actually translated into protein product, polyribosomes that were actively translating mRNA into protein were purified from K562 cells and Ex4a(+)WT1 mRNA associated with polyribosomes was analyzed by quantitative real-time PCR using 4a-forward and Ex6-reverse primer pair (**Fig 3F**). Ex4a(+)WT1 mRNA was detected in polyribosomes and the amount of Ex4a(+)WT1 mRNA in polyribosomes was significantly decreased by the addition of EDTA that released mRNA from polyribosomes. These results showed that endogenous Ex4a(+)WT1 mRNA existed in association with polyribosomes and thus was being translated into protein product.

### Expression of Ex4a(+)WT1 isoform in human cancer and normal cells

**Fig 4:**
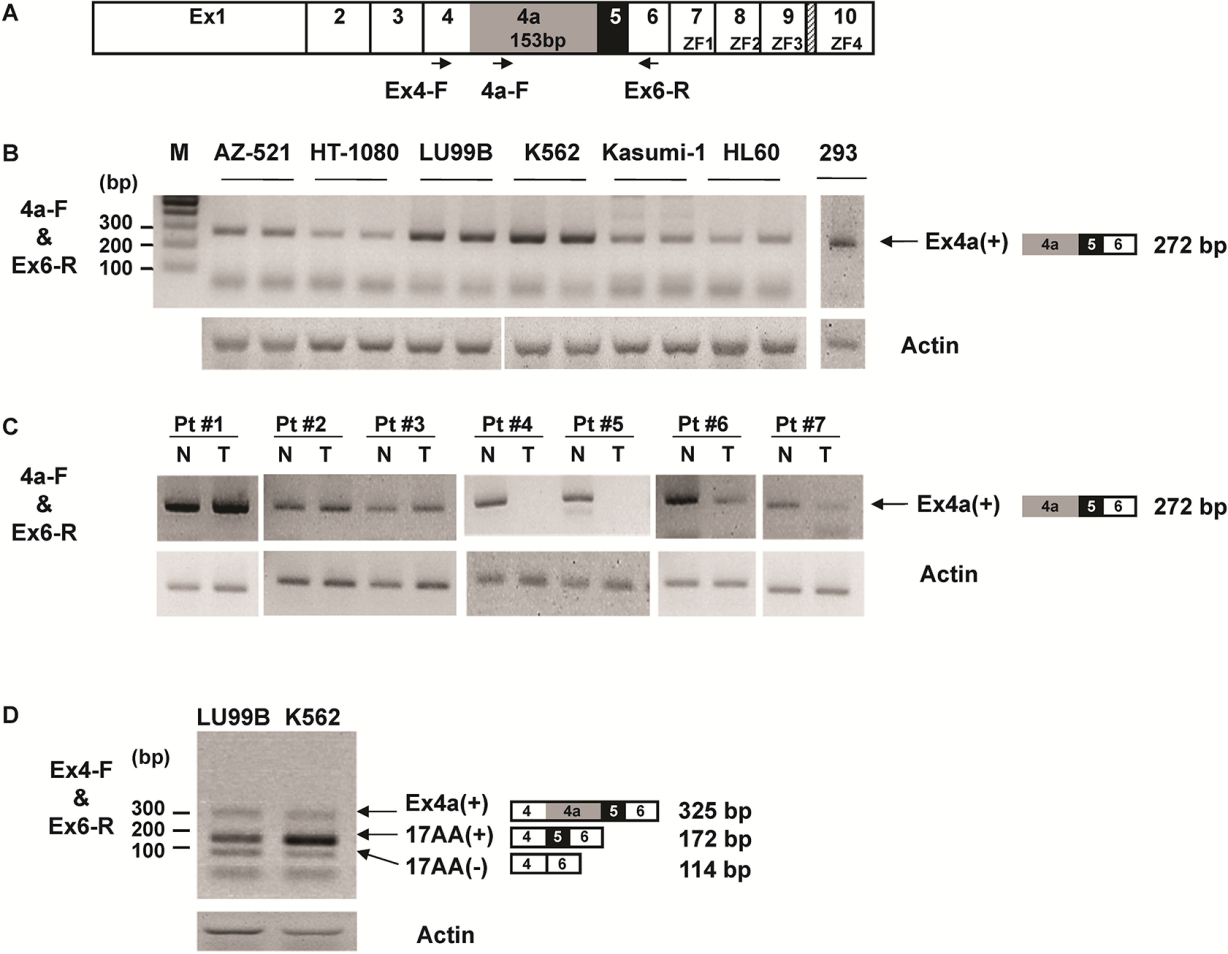
Expression of Ex4a(+)WT1 isoform in human cancer cells. (**A**) Schematic representation of the WT1 exons and localization of the primers used for semi-quantitative RT-PCR (arrows) are shown. (**B**) Ex4a(+)WT1 mRNA expression was determined by RT–PCR using 4a-F and Ex6-R primer pair that amplifies only Ex4a(+)WT1 isoform in six different WT1-expressing cancer cells (AZ-521, HT-1080, LU99B, K562, Kasumi-1 and HL60) and one WT1-expressiong normal kidney cells 293. (**C**) Ex4a(+)WT1 mRNA expression in the paired samples of tumor (T) and normal tissues (N) of seven NSCLC was determined by RT-PCR as indicated in (B). (**D**) The ratio of Ex4a(+)WT1 to 17AA(+)WT1 isoforms was determined by RT–PCR using Ex4-F and Ex6-R primer pair that amplifies both Ex4a(+)WT1 and major WT1 isoforms in two different WT1-expressing cancer cells (LU99B and K562). (B-D) Actin is used as an internal control. Results are representative of three independent experiments.

Ex4a(+)WT1 isoform expression in human cancer cells and normal kidney cells was examined (**Fig 4**). RT-PCR using 4a-forward and Ex6-reverse primer pair (**Fig 4A**) that amplified only the Ex4a(+)WT1 isoform was performed in six WT1-expressing cancer cell lines (AZ-521, HT-1080, LU99B, K562, Kasumi-1, and HL60) and one WT1-expressing normal kidney cell line 293 (**Fig 4B**). Ex4a(+)WT1 isoform was detected in all the WT1-expressing cancer and normal kidney cell lines. On the other hand, Ex4a(+)WT1 isoform was not detected in mouse WT1-expressing tissues such as kidney and testis (data not shown), consistent with low sequence identity in the novel alternative 5’ splice donor sequence between human and mouse as described in Fig 2D. Furthermore, seven paired samples of tumor and normal tissues of seven non-small cell lung cancer (NSCLC) was examined for the Ex4a(+)WT1 isoform (**Fig 4C**). The Ex4a(+)WT1 isoform was detected in all seven normal lung tissues and five of seven NSCLC tissues. Therefore, as for the Ex4a(+)WT1 isoform in normal cells, in three of seven paired samples, normal lung tissues expressed the Ex4a(+)WT1 isoform at levels comparable to those in lung cancer tissues. Interestingly, in the remaining four paired samples, normal lung tissues expressed the Ex4a(+)WT1 transcript higher than NSCLC tissues. These results indicated that the presence of the Ex4a(+)WT1 transcript was not to specific to tumor cells in which aberrant expression of various genes frequently occurred, and thus that the Ex4a(+)WT1 was physiologically transcribed.

Next, the ratio of Ex4a(+)WT1 isoform to 17AA(+) WT1 isoform was determined by RT-PCR using Ex4-forward and Ex6-reverse primer pair in WT1-expressing cancer cell lines (LU99B and K562) and determined to be approximately 1/2 and 1/4 in LU99B and K562 cancer cells, respectively (**Fig 4D**). These results indicated that the Ex4a(+)WT1 isoform was expressed as a minor isoform together with the major WT1 isoforms in cancer cells.

### Dominant negative function of an Ex4a(+)WT1 isoform

**Fig 5:**
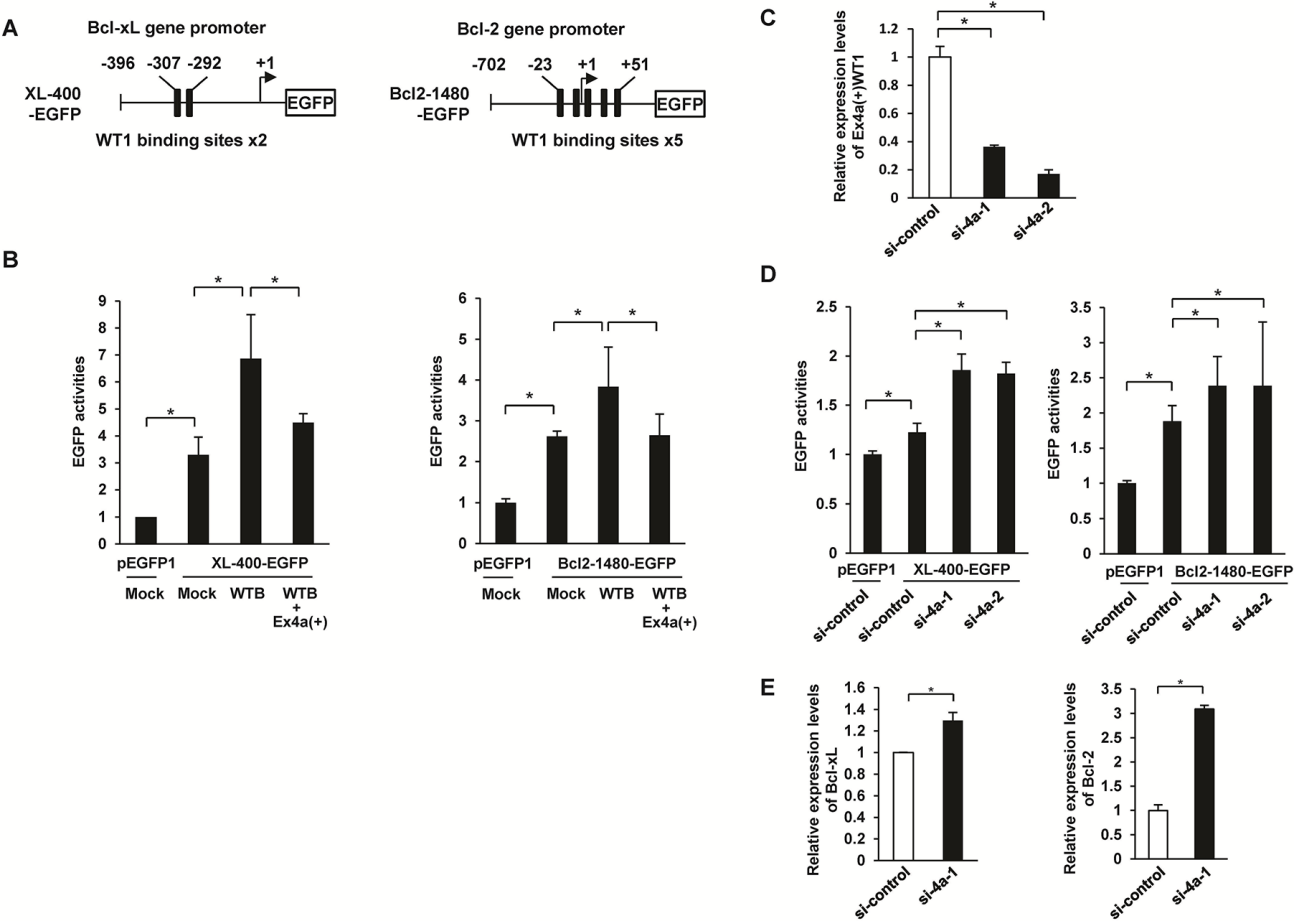
Dominant negative function of an Ex4a(+)WT1 isoform. (**A**) Schematic representation of the EGFP reporter vector containing 400-bp Bcl-xL promoter (XL-400-EGFP), 1,480-bp Bcl-2 promoter (Bcl2-1480-EGFP), and the potential WT1 binding sites are shown. (**B**) HT-1080 cells were co-transfected by 0.5 μg of XL-400-EGFP or 0.5 μg of Bcl2-1480-EGFP vector together with 1.5 μg of empty vector (Mock), 1.5 μg of 17AA(+)KTS(-)WT1 (WTB), or 1.5 μg of WTB plus 1.5 μg of Ex4a(+)WT1 vector. A CMV promoter-driven DsRed expression vector (0.5 μg) was co-transfected with each sample to normalize for differences in transfection efficiency. Appropriate amounts of empty vector (Mock) were added to each transfection mixture to make a total of 4.0 μg of plasmid DNA. EGFP activities were measured by flowcytometry after 48 h transfection. Relative mean fluorescence intensity (MFI) of EGFP is shown. MFI in promoterless pEGFP1 plus Mock-transfected cells are defined as 1.0. Results are means and S.D. of three independent experiments. *, p<0.05. (**C**) Knockdown of Ex4a(+)WT1 isoform expression by siRNAs. K562 cells were transfected with either of two different Ex4a-specific siRNAs (si-4a-1 and si-4a-2) or control siRNA (si-control) for 48 h and Ex4a(+)WT1 mRNA expression was determined by quantitative real-time RT-PCR using 4a-F and Ex6-R primer pair. Actin is used as an internal control for normalization. Expression levels of Ex4a(+)WT1 in si-control-transfected cells are defined as 1.0. Results are means and S.D. of three independent experiments. *, p<0.05. (**D**) K562 cells were co-transfected by 5.0 μg of XL-400-EGFP or 5.0 μg of Bcl2-1480-EGFP reporter vector together with one μg of either of two WT1 Ex4a-specific siRNAs (si-4a-1 and si-4a-2) or control siRNA (si-control). A CMV promoter-driven DsRed expression vector (0.5 μg) was co-transfected with each sample to normalize for differences in transfection efficiency. EGFP activities were measured by flowcytometry 48 h after transfection. MFI of EGFP is shown. MFI in promoterless pEGFP1 vector plus si-control-transfected cells are defined as 1.0. Results are means and S.D. of three independent experiments. *, p<0.05. (**E**) K562 cells were transfected with either of two different Ex4a-specific siRNAs (si-4a-1 and si-4a-2) or control siRNA (si-control) for 48 h and Bcl-xL and Bcl-2 mRNA expression were determined by quantitative real-time RT-PCR. Actin is used as an internal control for normalization. Expression levels in Mock-transfected cells are defined as 1.0. Results are means and S.D. of three independent experiments. *, p<0.05.

Dominant negative effect of the Ex4a(+)WT1 on the function of major WT1 isoforms was examined. *Bcl-xL* promoter contains two WT1 binding sites 5’-GCGGGGGAGC-3’ and 5’-GAGCGGGAGT-3’, which are similar to the consensus WTE motif 5'-GCGTGGGAGT-3' [12] at positions −307 to −298 and -301 to -292 upstream of the transcription start site of *Bcl-xL* gene (**Fig 5A left**). *Bcl-2* promoter also contains five WT1 binding sites with the consensus sequence 5’-GNGNGGGNG-3’ [11] (**Fig 5A right**). Previous studies showed that wild-type major WT1 isoform directly binds to these two promoters and activates their transcription [14, 15]. On the basis of these findings, effects of Ex4a(+)WT1 isoform on the wild-type major WT1 isoform-mediated transcriptional activation of *Bcl-xL* and *Bcl-2* genes were examined. When 400-bp *Bcl-xL* promoter-EGFP reporter vector (XL-400-EGFP) or 1,480-bp *Bcl-2* promoter-EGFP (Bcl2-1480-EGFP) reporter vector was co-transfected with 17AA(+)KTS(-)WT1 isoform (WTB) into HT-1080 cells, EGFP activities increased. However, the activation of XL-400-EGFP and Bcl2-1480-EGFP by WTB was reduced by co-transfection of Ex4a(+)WT1 isoform (**Fig 5B left**). These results indicated that Ex4a(+)WT1 isoform dominant negatively suppressed transcriptional activation of *Bcl-xL* and *Bcl-2* genes by major WT1 isoform(s).

The involvement of the endogenously expressed Ex4a(+)WT1 in the transcriptional regulation of *Bcl-xL* and *Bcl-2* genes was examined by knockdown of Ex4a(+)WT1 expression using two different kinds of WT1 Ex4a-specific siRNAs. XL-400-EGFP or Bcl2-1480-EGFP reporter vector was co-transfected with either of two different WT1 Ex4a-specific siRNAs (si-4a-1 and si-4a-2) or a control siRNA (si-control) into K562 cells and then EGFP activities were analyzed. Both of two WT1 Ex4a-specific siRNAs (si-4a-1 and si-4a-2) decreased Ex4a(+)WT1 mRNA expression (**Fig 5C**) and increased the EGFP activities of XL-400-EGFP and Bcl2-1480-EGFP reporter vectors (**Fig 5D left**). Furthermore, transfection of WT1 Ex4a-specific siRNA (si-4a-1) increased the endogenous Bcl-xL and Bcl-2 mRNA expression (**Fig 5E**). These results indicated that endogenously expressed Ex4a(+)WT1 transcript had physiological function to regulated the transcriptional activities of *Bcl-xL* and *Bcl-2* genes.

The dominant negative suppression of transcriptional activity of major WT1 isoform by Ex4a(+)WT1 isoform raised the possibility of physical association between the major WT1 and Ex4a(+)WT1 isoforms. To test the possibility, the cells that fully expressed Ex4a(+)WT1 isoform together with major WT1 isoforms were first established (**Figure A in S2 Fig**), and then immune-precipitation assay was performed using anti-WT1 antibody (C-19) against C-terminal portion of WT1 protein or control non-immune IgG. Immunoprecipitation of major WT1 isoforms with C-19 antibody but not with control non-immune IgG was confirmed. However, Ex4a(+)WT1 isoform was not co-precipitated with major WT1 isoform using C-19 antibody (**Figure B in S2 Fig**). Therefore, it appeared that Ex4a(+)WT1 isoform did not physically associate with major WT1 isoforms.

### Apoptotic function of an Ex4a(+)WT1 isoform

**Fig 6:**
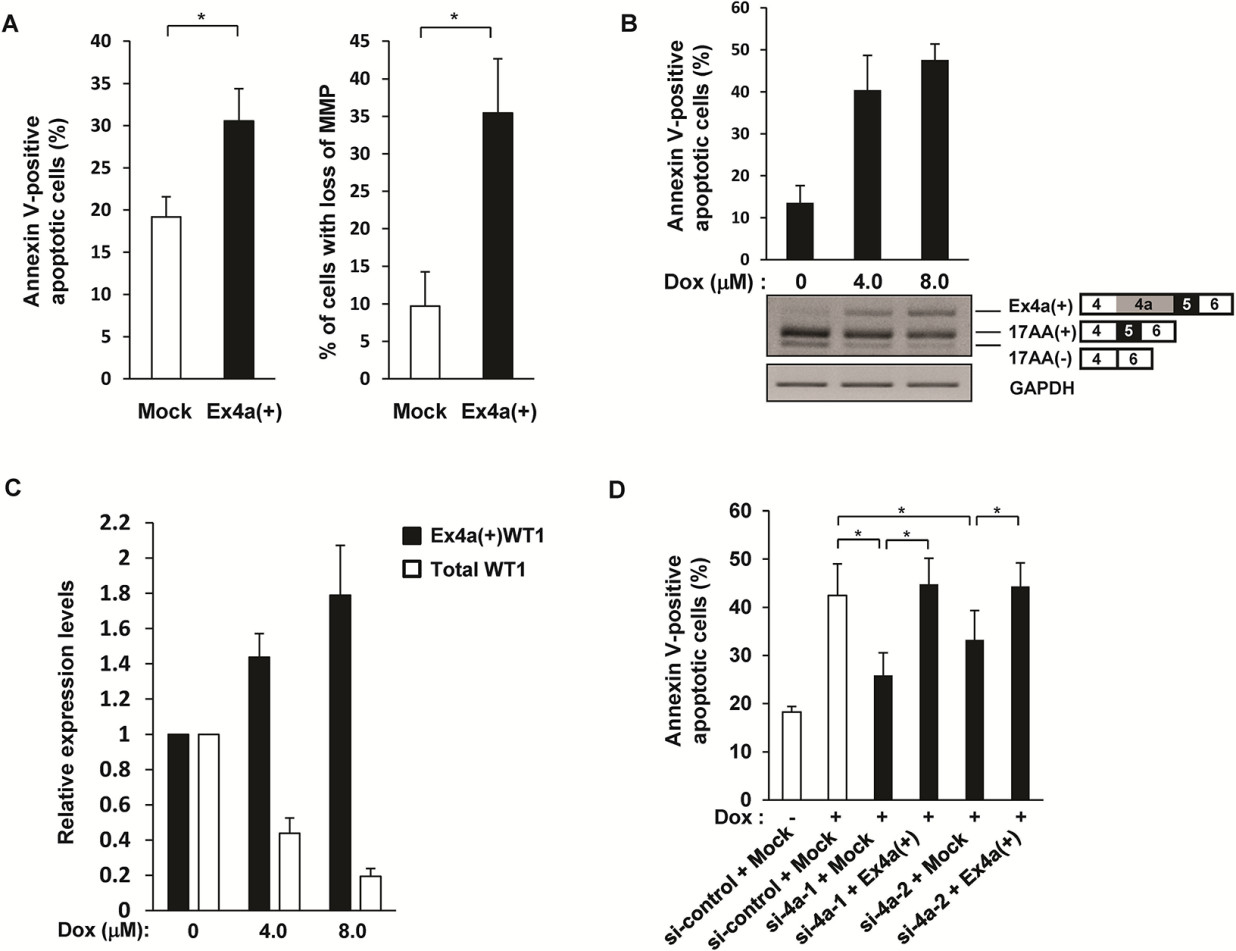
Apoptotic function of an Ex4a(+)WT1 isoform. (**A**) The role of Ex4a(+)WT1 in apoptosis. Ex4a(+) or Mock vector was transfected into WT1-expressing HT-1080 cells. Frequencies (%) of Annexin V-positive apoptotic cells and cells with loss of MMP were determined by flowcytometry after 24 h. Left, Frequencies (%) of Annexin V-positive apoptotic cells are shown. Right, Frequencies (%) of cells with mitochondrial membrane potential (MMP) loss are shown. Results are means and S.D. of three independent experiments. *, p<0.05. (**B**) Expression of Ex4a(+) and major WT1 isoforms during apoptosis. K562 cells were treated with the indicated concentrations of Dox for 12 h and analyzed for Annexin-V positive apoptotic cells and expression of Ex4a(+) and major WT1 isoforms by flowcytometry and RT-PCR, respectively. Upper, Frequencies (%) of Annexin V-positive apoptotic cells. Lower, RT-PCR using Ex4-F and Ex6-R primer pair that amplifies both Ex4a(+) and major WT1 isoforms. GAPDH is used as an internal control. Results are representative of three independent experiments. (**C**) Change of Ex4a(+)WT1 and major WT1 isoforms during apoptosis. K562 cells were treated with the indicated concentrations of Dox for 12 h and expression of Ex4a(+)WT1 and total WT1 isoforms including both Ex4a(+) and major WT1 isoforms were determined by quantitative real-time RT-PCR using Ex4a-F and Ex6-R primer pair and Ex6-F and Ex7-R primer pair, respectively. Actin is used as an internal control for normalization. Expression levels of Ex4a(+)WT1 and total WT1 in Dox-untreated cells are defined as 1.0. (**D**) Suppression of Ex4a(+)WT1 inhibits Dox-induced apoptosis. K562 cells were transfected with one μg of either of two WT1 Ex4a-specific siRNAs (si-4a-1 and si-4a-2) or a control siRNA (si-control) together with 2.0 μg of Ex4a(+)WT1 vector or 2.0 μg of empty vector (Mock), cultured for 24 h, treated with 4.0 μM Dox for 12 h, and then analyzed for Annexin-V positive apoptotic cells by flowcytometry. Frequencies (%) of Annexin V-positive apoptotic cells are shown. Results are mean and S.D. of three independent experiments. *, p<0.05.

It is well known that major isoforms of WT1 17AA(+) and 17AA(-) have an anti-apoptotic function. Therefore, the role of the Ex4a(+)WT1 isoform in apoptosis was examined (**Fig 6**). HT-1080 cells were transfected with Ex4a(+)WT1 isoform or Mock, and analyzed for apoptosis and mitochondrial damages (**Fig 6A**). Ex4a(+)WT1 isoform significantly induced apoptosis and mitochondrial membrane potential (MMP) loss in HT-1080 cells.

Next, the role of the endogenous Ex4a(+)WT1 isoform in apoptosis was examined. K562 cells were treated with Doxorubicin (Dox) and apoptosis was induced. Then, Ex4a(+)WT1 and major WT1 isoforms were determined by RT-PCR using Ex4-forward and Ex6-reverse primer pair described in Fig 4A (**Fig 6B**). Major WT1 isoform was decreased by apoptosis, whereas Ex4a(+)WT1 isoform was increased. Furthermore, the Ex4a(+)WT1 isoform and total WT1 isoforms including both Ex4a(+) and major WT1 isoforms were quantified by quantitative real-time PCR (**Fig 6C**). The results confirmed that Ex4a(+)WT1 isoform was increased and major WT1 isoforms were decreased by apoptosis. When etoposide was used as an apoptosis inducer, the similar results were obtained (data not shown). These results indicated that increased expression of the Ex4a(+)WT1 isoform might contribute to the induction of apoptosis.

Moreover, to examine the effects of the endogenous Ex4a(+)WT1 isoform on Dox-induced apoptosis, K562 cells were transfected with either of two different WT1 Ex4a-specific siRNAs (si-4a-1 and si-4a-2) or control siRNA (si-control) for 24 h, treated with Dox for 12 h, and then analyzed for apoptosis (**Fig 6D**). Transfection of both of two WT1 Ex4a-specific siRNAs (si-4a-1 and si-4a-2) resulted in significant decrease in Dox-induced apoptosis. Furthermore, the reduction in Dox-induced apoptosis by Ex4a-specific siRNAs was cancelled by concomitant forced overexpression of Ex4a(+)WT1 isoform. These results indicated that endogenous Ex4a(+)WT1 had a physiological function to promote apoptosis.

## Discussion

The *WT1* gene produces four major isoforms by alternative splicing of exon 5 (17AA) and exon 9 (KTS). All four major isoforms are overexpressed in leukemia and solid tumors and play oncogenic roles in tumorigenesis and leukemogenesis. In addition to the four major splicing isoforms, N-terminal extended and truncated WT1 isoforms have been previously identified. Translation initiation at an upstream CUG and downstream AUG codons produce larger N-terminal extended WT1 with an additional 68AA and smaller N-terminal truncated WT1 lacking the first 126AA, respectively [25, 26]. Transcription initiation at intron 1 and intron 5 produce N-terminal truncated WT1 lacking the first 147AA (known as sWT1) and lacking exons 1 to 5, respectively [28–30]. All these known WT1 isoforms contain an intact zinc finger domain in their C-terminus. It has been shown that the N-terminal truncated sWT1 isoform is more effective in the transcriptional activation of downstream targets because of the lack of repressor domain of N-terminus and thus exhibits more oncogenic potential than major WT1 isoforms [28].

On the other hand, the present study for the first time identified a C-terminal truncated WT1 splice isoform lacking entire zinc finger domain, designated as an Ex4a(+)WT1 isoform, which was produced by an natural alternative splicing mechanism from wild WT1 genome without mutations. The major WT1 isoforms are known to have an anti-apoptotic function. By contrast, the C-terminal truncated Ex4a(+)WT1 isoform had a pro-apoptotic function, probably through suppression of anti-apoptotic function of major WT1 isoforms. The identification of the pro-apoptotic Ex4a(+)WT1 isoform should be helpful for better understanding of the mechanism of WT1-mediated leukemogenesis and tumorigenesis.

In this study, Ex4a(+)WT1 isoform inhibited the transcriptional activation of *Bcl-xL* and *Bcl-2* genes by major WT1 isoform [17AA(+)KTS(-)WT1]. It was reported that major WT1 isoform exerts transcriptional activity through the interaction between the N-terminal domain of WT1 protein and transcriptional co-activators including WT1 itself [8, 37]. The C-terminal truncated Ex4a(+)WT1 isoform contained the N-terminal transcriptional regulatory domain but lacked the zinc finger DNA-binding domain, and thus the truncated Ex4a(+)WT1 isoform retained the ability to interact with the transcriptional co-activators but lost the ability to bind to promoter of downstream targets. Previous studies have shown that the N-terminal domain of WT1 (a.a. 1–182 and 1–326) lacking zinc finger inhibit the major WT1 isoform-mediated transcriptional regulation through self-association in yeast cells and human osteosarcoma cells [7, 38]. On the other hand, the Ex4a(+)WT1 isoform did not physically associate with major WT1 isoforms. Therefore, it was reasonable that the mechanism of inhibition of major WT1 isoform-mediated transcriptional activation of *Bcl-xL* and *Bcl-2* by Ex4a(+)WT1 isoform might be competition with major WT1 isoform for interacting with the transcriptional co-activators other than WT1 itself, resulting in the abrogation of major WT1 isoform-mediated transcriptional activity in leukemia cells. However, precise molecular mechanism of suppressive effects of Ex4a(+)WT1 on major WT1 isoform-mediated transcriptional activation remains undetermined at present time, and elucidation of the mechanism should be interesting.

The suppression of major WT1 isoform-mediated transcriptional activation by Ex4a(+)WT1 might be extended to other direct transcriptional targets as well as *Bcl-xL* and *Bcl-2*. Since some other genes responsible for anti-apoptosis such as *BFL-1* and *c-myc* have been also identified as direct transcriptional targets of major WT1 isoforms [16, 17], Ex4a(+)WT1-induced apoptosis might be caused by competition with major WT1 isoform for binding to promoter of these transcriptional targets, resulting in the decrease in their expression.

The present study showed that the *WT1* gene was alternatively spliced at Ex4a to produce isoforms with opposing roles in apoptosis, anti-apoptotic isoforms (major WT1 isoforms) and pro-apoptotic isoform (truncated Ex4a(+)WT1 isoform). Several other genes involved in apoptosis, such as *p53* [39], *Survivin* [40], *Fas* [41], and *caspase-9* [42] are known to produce isoforms with opposing roles in promoting or inhibiting apoptosis by alternative splicing. In all these cases, alternatively spliced truncated isoform regulates apoptosis through inhibition of the function of wild-type protein by a dominant negative mechanism. For example, alternatively spliced truncated p53 isoform termed p47 inhibits wild-type p53-induced apoptosis through suppression of wild-type p53-mediated transcriptional activity [39]. Truncated Survivin-2a isoform attenuates the anti-apoptotic activity of wild-type Survivin, possibly through direct interaction with wild-type Survivin [40]. These examples, together with our present results, suggested that production of truncated isoforms with opposing functions from a single gene was a critical regulatory mechanism in apoptosis.

## Materials and Methods

### Cell lines and cultures

Chronic myeloid leukemia K562 [43], acute myeloid leukemia Kasumi-1 [44], and acute promyelocytic leukemia HL60 [45] cell lines were cultured in RPMI1640 medium supplemented with 10% FBS. Lung cancer LU99B [46], gastric cancer AZ-521 [47], fibrosarcoma HT-1080 [48] and human embryonic kidney 293 [49] cell lines were cultured in Dulbecco’s modified essential medium supplemented with 10% FBS. All cell lines used in this study were obtained from Japan Health Sciences Foundation (Osaka, Japan).

### Chemical reagents

Doxorubicin (Dox, Sigma Chemical Co., Steinheim, Germany) and Etoposide (WAKO, Osaka, Japan) were used to induce apoptosis. Cells were grown to 80% confluence, treated with Dox at the indicated concentrations for 12 h, and then harvested. Puromycin (Invitrogen, Carlsbad, CA) was used to block nonsense mediated mRNA decay (NMD) pathway [50]. Cells were grown to 80% confluence, treated with 100 μg/ml puromycin for 4 h, and then harvested. Actinomycin D (ActD, Sigma Chemical Co., Steinheim, Germany) was used to inhibit transcription. Cells were grown to 80% confluence, treated with 5 μg/ml of ActD, and then harvested at indicated time points.

### Cloning of the full-length Ex4a(+)WT1 cDNA

To obtain the 3’ end of the Ex4a(+)WT1 cDNA, 3’ rapid amplification of cDNA ends (3’ RACE) was performed. Total RNA from K562 cells was reverse-transcribed with 3’ RACE adapter-dT primer (5’-GCGAGCACAGAATTA

ATACGACTCACTATAGG-(T)_12_VN-3’) and M-MLV reverse transcriptase (Promega, Madison, WI). First PCR was performed by using exon 4a-specific forward primer (4a-F, 5’-ATTCCATTGCCTTTCCACAG-3’) and 3' RACE outer reverse primer (5’-GCGAGCACAGAATTAATACGACT-3’). Nested PCR was performed by using the first PCR product as a template with the nested Ex4a(+)WT1 cDNA-specific forward primer (4a-F2, 5’-GCAAAGCTCTCTGTG

ACATTA-3’) and the nested adaptor inner primer (5’-CGCGGATCCGAATTAA TACGACTCACTATAGG-3’). The PCR reactions with Ex-Taq polymerase (Takara, Shiga, Japan) were as follows: 94°C, 30 sec; 40°C, 30 sec; and 72°C, 60 sec for 5 cycles, followed by 94°C, 30 sec; 50°C, 30 sec; and 72°C, 60 sec; for 35 cycles. The second PCR products were cloned into pCR2.1 TA vector (Invitrogen, Carlsbad, CA) and sequenced.

To obtain the 5’ end of the Ex4a(+)WT1 cDNA, the WT1-specific primers were designed upstream (F1, F2, F3, and F4) and downstream (F5, F6, and F7) of the major transcriptional start site of WT1 that was registered in Database of Transcriptional Start Sites (DBTSS, http://dbtss.hgc.jp/, [33]) (Fig 2A). Total RNA from K562 cells was reverse-transcribed with oligo-(dT) primer and M-MLV reverse transcriptase (Promega, Madison, WI). PCR was performed by using one each of seven forward primers (F1-F7) and exon 4a-specific reverse primer (Ex4a-R). The PCR reactions with KOD-FX (Toyobo, Osaka, Japan) were as follows: 98°C, 10 sec; 60°C, 30 sec; and 68°C, 60 sec for 35 cycles. The PCR products amplified by using F5 and 4a-R were cloned into pCR-BluntII-TOPO vector (Invitrogen, Carlsbad, CA) and sequenced. Primer sequences used to obtain the 5’ end were as follows: F1, 5’-CCGGCTTATAACTGGTGCAAC-3’; F2, 5’-ACGGACTCTCCAGTGAGACG-3’; F3, 5’-GGCTGCTGAGTGAATGGAG-3’; F4, 5’-CCCCTCTTATTTGAGCTTTGG-3’; F5, 5’-CCAGGCAGCTGGGGTAAG

GAGTTCA-3’; F6, 5’-TCCTGGACTTCCTCTTGCTG-3’; F7, 5’-ATGGGCTCCG

ACGTGCGGGACCTGAAC-3’; 4a-R, 5’-CTGTGGAAAGGCAATGGAAT-3’.

The Ex4a(+)WT1 cDNA sequence described in this work has been deposited in GenBank/EMBL/DDBJ under the accession No: AB971668.

### Semi-quantitative RT-PCR

Total RNA was isolated using TRIZOL (Invitrogen, Carlsbad, CA) and reverse-transcribed using oligo (dT)_18_ primers and M-MLV reverse transcriptase (Promega, Madison, WI) according to the manufacturer’s instructions. The PCR was performed using Ex-Taq polymerase (TaKaRa, Shiga, Japan) by the following conditions: 94°C, 60 sec; 60°C, 60 sec; 72°C, 60 sec for 35 cycles. For the amplification of both the Ex4a(+) and major WT1 [17AA(+) and 17AA(-)WT1] isoforms, primer pair of Ex4-F and Ex6-R was used. For the amplification of Ex4a(+)WT1 isoform, primer pair of 4a-F and Ex6-R was used. Sequences of the primers for semi-quantitative PCR were as follows; Ex4-F, 5’-GACCTGGAAT

CAGATGAACTTAG-3’, 4a-F, 5’-ATTCCATTGCCTTTCCACAG-3’; Ex6-R, 5’-GA

CACCGTGCGTGTGTATTC-3’, Ex9-R, 5’-GAGAACTTTCGCTGACAAGTT-3’; Actin-F, 5’-CCCAGCACAATGAAGATCAAGATCAT-3’; Actin-R, 5’-ATCTGCTGG

AAGGTGGACAGCGA-3’; GAPDH-F, 5’-GCCAAAAGGGTCATCATCTC-3’; GAPDH-R, 5’-GTAGAGGCAGGGATGATGTTC-3’.

### Quantitative real-time RT-PCR

Quantitative RT-PCR (qPCR) assays were run on a Chromo4 system (BIO-RAD) using GoTaq SYBR Green PCR kit (Promega, Madison, WI). Each reaction was performed in a final volume of 25 μl containing one μl cDNA and 2 x SYBR Green PCR Master mix. The amplification profile was denaturation at 94°C for 2 min, followed by 40 cycles of 94°C for 10 sec, 58°C for 30 sec. At the end of the PCR cycles, melting curve analysis of the PCR products was performed to validate the amplification of the specific products. Primer pair of 4a-F and Ex6-R for Ex4a(+)WT1 amplification and Ex6-F and Ex7-R primer pair for total WT1 amplification were used, respectively. The expression levels of WT1 mRNA were calculated using the comparative Ct method (2^-ΔΔ^Ct) with Actin as the reference gene. Primer sequences for qPCR are as follows: 4a-F, 5’-ATTCCATTGCCTTTC

CACAG-3’; Ex6-R, 5’-GACACCGTGCGTGTGTATTC-3’; Ex6-F, 5’-GATAACCAC

ACAACGCCCATC-3’; Ex7-R, 5’-CACACGTCGCACATCCTGAAT-3’; Actin-F, 5’-CCCAGCACAATGAAGATCAAGATCAT-3’; Actin-R, 5’-ATCTGCTGGAAGGT

GGACAGCGA-3’. Bcl-xL-F, 5’-GATCCCCATGGCAGCAGTAAAGCAAG-3’; Bcl-xL-R, 5’-CCCCATCCCGGAAGAGTTCATTCACT-3’; Bcl-2-F, 5’-CGCCCTGT

GGATGACTGAG-3’; Bcl-2-R, 5’-AGCCAGGAGAAATCAAACAGAGG-3’. MRP4-F, 5’-AGGACACTTGCCATTGGATTA-3’; MRP4-R, 5’-ACCCTTGCAACT

CCTCTCCAAG-3’.

### Clinical samples

Lung cancer tissues were obtained from patients who underwent curative resection for lung cancer at National Hospital Organization Kinki-Chuo Chest Medical Center. All these samples were collected with written informed consent and approved by Ethical Review Board of School of Allied Health Science, Osaka University, Faculty of Medicine, Osaka University (#13–1).

### Mouse tissues

Six-week-old male C57BL/6 mice (SLC, Shizuoka, Japan) were used for this study. They were maintained in accordance with the guidelines for animal experiments of Osaka University.

### Plasmid construction

WT1 fragment from exons 1 to 4a expression vector (Ex4a(+)), N-terminal His-tagged full-length Ex4a(+)WT1 (His-Ex4a(+)full) and WT1 fragment from exons 1 to 4 (His-Ex1-4) expression vectors were constructed. To construct Ex4a(+) and His-Ex1-4 vectors, WT1 cDNA fragment from exons 1 to 4a and exons 1 to 4 were PCR-amplified and inserted into EcoRI site of pcDNA3.1 (Ex4a(+)) or pcDNA3.1-His vector (His-Ex1-4), respectively (Invitrogen, Carlsbad, CA). To construct His-Ex4a(+)full expression vector, WT1 cDNA fragments from exons 1 to 4a and exons 5 to 10 were PCR-amplified and inserted into EcoRI/NotI and NotI/XbaI site, respectively, of pcDNA3.1-His vector. 17AA(+)KTS(-)WT1 isoform (WTB) vector and N-terminal His-tagged 17AA(+)KTS(+)WT1 isoform (His-WTD) vector were previously constructed [22]. K562 cell clones that stably expressed the His-Ex4a(+)full were isolated using G-418 at the concentration of 700 μg/ml.

Bcl-xL and Bcl-2 promoter-EGFP reporter vectors were constructed as follows. Bcl-xL promoter of 400 bp (positions -396 to +33 relative to the transcriptional start site) was PCR-amplified from human genomic DNA, cloned into BglII/PstI site upstream of the EGFP coding gene of pEGFP-1 vector (Clontech, Palo Alto, CA) and designated as XL-400-EGFP. Bcl-2 promoter of 1840 bp (positions –702 to + 781 relative to the transcriptional start site) was PCR-amplified from human genomic DNA, cloned into EcoRI/BamHI site upstream of the EGFP coding gene of pEGFP-1 vector and designated as Bcl2-1840-EGFP. The primer sequences for Bcl-xL and Bcl-2 promoter amplification were as follows: XL-400-forward, 5’-CCACCTCCTCTCCCGACCTGTGATACAAAAGAT-3’; XL-400-reverse, 5’-ATTCTGCAGCACTCACTGAGTCTCGTCTCTGGT-3’. Bcl2-1480-forward, 5’-GCGCGTGTACACACTCTCAT-3’; Bcl2-1480-reverse, 5’-TTCCAGATCGATTCCCAGAC-3’.

### Plasmid transfection using polyethylenimine (PEI)

For transient expression, cells were transfected with plasmid DNA using 25-kDa linear polyethylenimine (PEI, Polysciences) at a PEI/DNA ratio of 20:1 [51].

### Small interfering RNA (siRNA) and transfection

To knockdown Ex4a(+)WT1 isoform expression, two different kinds of WT1 exon 4a-specific siRNAs (si-4a-1; sense sequence, GUGCUUAGAACUUAUGAAGAA

dTdT; antisense sequence, UUCUUCAUAAGUUCUAAGCACdTdT and si-4a-2; sense sequence, GCCUUUCCACAGUAACUUAUAdTdT; antisense sequence, UAUAAGUUACUGUGGAAAGGCdTdT) were used (GeneDesign, Osaka, Japan). A scrambled sequence (si-control) was used as a negative control.

Cells (5x10^5^ cells) were transfected with one μg of either si-4a-1, si-4a-2, or si-control by electroporation (155 V, 1000 μFD) using Gene Pulsor II (BioRad, CA, USA).

### EGFP-promoter reporter assay

HT-1080 cells were co-transfected by 0.5 μg of XL-400-EGFP or 0.5 μg of Bcl2-1480-EGFP together with 1.5 μg of empty vector (Mock), 1.5 μg of 17AA(+)KTS(-)WT1 (WTB), or 1.5 μg of WTB plus 1.5 μg of Ex4a(+)WT1 vector. pIRES2-DsRed-Express2 vector (Clontech, Palo Alto, CA) for expression of DsRed gene was also included in each transfection mixture as an internal control to normalize for differences in transfection efficiency. Appropriate amounts of empty vector (Mock) were added to each transfection mixture to make a total of 4.0 μg of plasmid DNA. EGFP activities were measured by flowcytometry after 48 h transfection.

K562 cells (1.5 x 10^5^ cells) were co-transfected by 5.0 μg of promoter-EGFP vector together with one μg of either of two WT1 Ex4a-specific siRNAs (si-4a-1 and si-4a-2) or control siRNA (si-control) by electroporation (155 V, 1000 μFD) using Gene Pulsor II (BioRad, CA, USA), harvested after 48 h, and analyzed for EGFP activities by flowcytometry.

### Western blot analysis

Cells were lysed, and proteins were separated by SDS-PAGE and transferred to PVDF membrane. After blocking of non-specific binding, immunoblots were incubated with monoclonal antibodies against the N-terminal region (a.a. 1–181) of WT1 protein (6F-H2, Dako Cytomation, Carpinteria, CA), GAPDH (6C5, Millipore, Temecula, CA) and His-tag (Anti-Xpress antibody, Invitrogen, Carlsbad, CA) or polyclonal antibody against the C-terminal region (a.a. 431–450) of WT1 protein (C-19, Santa Cruz Biotechnology), followed by incubation with an anti-mouse or rabbit IgG antibody conjugated with alkaline phosphatase (Santa Cruz Biotechnology), and visualized using BCIP/NBT kit (Nacalai Tesque, Kyoto, Japan).

### Isolation of nuclear and cytoplasmic protein

K562 cells were washed with PBS, lysed in 1.0 ml of PBS containing 10% Triton-X-100 on ice for 30 min, and then centrifuged at 12,000 g at 4°C for 15 min. The pellet was solved in SDS sample buffer and stored as a nuclear fraction. The proteins in the supernatant was precipitated with acetone, solved in SDS sample buffer and stored as a cytoplasmic fraction.

### Immunoprecipitation assay

K562 cells stably transduced with His-tagged Ex4a(+)WT1 [K562-His-Ex4a(+)] were washed with ice-cold PBS, lysed in RIPA buffer (50mM Tris-HCl pH8.0, 150 mM NaCl, 0.5% NP40, 0.5% deoxycholate, 1mM EDTA) containing 1 mM PMSF, incubated on ice for 20 min, and then centrifuged at 12.000 x g for 20 min at 4ºC. The supernatants were collected as cell lysates. The cell lysates were precleared with Protein G-sepharose beads (GE Healthcare) for 30 min and then incubated overnight at 4ºC with 3 μg of C-19 or non-immune rabbit IgG coupled to Protein G–sepharose beads. The immunoprecipitates were washed with RIPA buffer and proteins were eluted by boiling in SDS sample buffer. The eluted proteins were separated by SDS-PAGE and analyzed with 6F-H2 antibody (Dako Cytomation, Carpinteria, CA) against the N-terminal region of WT1 protein.

### Polyribosome purification

Ex4a(+)WT1 mRNA associated with polyribosomes was analyzed as described previously with minor modifications [52, 53]. In brief, 5x10^6^ K562 cells were washed with ice-cold PBS and lysed in 1.5 ml of polyribosomal buffer (25 mM Tris [pH 8.0], 50 mM NaCl, 5 mM MgCl_2_, 250 mM sucrose, 200 U of RNase inhibitor per ml, and 1% Triton X-100) on ice for 30 min. After removing the nuclei, mitochondria, and cell debris by centrifugation at 12,000 g at 4°C for 15 min, the supernatant was ultracentrifuged at 130,000 g at 4°C for 12 min (Beckman Coulter, OptimaTLX) and the resulting pellets (polyribosomes) were resuspended in TRIZOL. The polyribosomal RNAs were reverse-transcribed with oligo-(dT) primer and M-MLV reverse transcriptase and subjected to real-time PCR analysis of Ex4a(+)WT1 mRNA. As a control, an EDTA release experiment was performed as described previously [53], where the polyirbosomal buffer was supplemented with 100 mM EDTA (pH 8.0) to release mRNA from ribosome.

### Analysis of apoptosis and mitochondrial membrane potential

Apoptotic cells were assessed using MEBCYTO Apoptosis Kit (MBL Co., Ltd, Aichi, Japan) according to the manufacturer’s instructions. In brief, 1x10^5^ cells were washed with PBS, and stained with Annexin V-fluorescein isothiocyanate (FITC) and propidium iodide (PI) at room temperature for 15 min in the dark. Then, the stained cells were analyzed by a FACSCalibur flowcytometer (Becton Dickinson, San Jose, CA).

Changes in mitochondrial membrane potential (MMP) were assessed using MitoLight apoptosis detection kit (Chemicon International, Temecula, CA) according to the manufacturer’s instructions. In brief, 1x10^5^ cells were incubated in MitoLight dye solution that stained mitochondria in living cells at 37°C for 15 min. Then, a red fluorescence yielded was analyzed by a FACSCalibur flowcytometer (Becton Dickinson, San Jose, CA).

### Statistical analyses

Statistical analysis was performed by unpaired Student's t test or one-way ANOVA followed by Tukey's multiple comparison test. The data were presented as the mean and standard deviation (S.D.) from more than three independent experiments. P-values < 0.05 were considered to be statistically significant.

## Supporting Information

S1 Fig Endogenous truncated Ex4a(+)WT1 proteins could not be detected by SDS-PAGE analysis. (**A**) Nuclear and cytoplasmic fractions of K562 cells were isolated and then examined for WT1 protein expression by Western blot analysis with 6F-H2 (specific for the N-terminal region of WT1 protein) antibody. N and C indicate the nuclear and cytoplasmic fractions of K562 cells, respectively. W indicates whole cell lysate from K562 cells. (**B**) WT1 protein expression was examined in Dox-treated K562 cells, where Ex4a(+)WT1 mRNA increased. K562 cells were treated with 4 μM of Dox for 24 h and analyzed for WT1 protein expression by Western blot analysis with 6F-H2 antibody (Left). (**A-B**) Results are representative of three independent experiments.
(TIF)

S2 Fig No physical interaction between the Ex4a(+)WT1 and major WT1 isoforms. (**A**) Establishment of K562 cell clones transduced with His-tagged Ex4a(+)WT1 isoform. Expression of His-Ex4a(+)WT1 proteins were examined by Western blot analysis with anti-His tag (Left) or 6F-H2 (specific for the N-terminal region of WT1 protein) (Right) antibody. MW represents molecular weight marker. Arrowheads and arrows indicate major WT1 protein isoforms and 30-KDa His-tagged truncated Ex1-4 WT1 protein, respectively. (**B**) Immunoprecipitation assay. Cell lysates from K562-His-Ex4a(+)WT1 cells were subjected to immunoprecipitation with C-19 (specific for the C-terminal region of WT1 protein) antibody or control non-immune IgG (IgG). The resulting immunoprecipitated complexes were separated by SDS-PAGE and analyzed with 6F-H2 (specific for the N-terminal region of WT1 protein) antibody. MW represents molecular weight marker. Arrowheads and arrows indicate major WT1 protein isoforms and 30-KDa His-tagged truncated Ex1-4 WT1 protein, respectively. Cell lysates are immunoblotted as a control (Extract). Results are representative of three independent experiments.
(TIF)
