# Supplementary figures and images for: Identification of a Novel C-Terminal Truncated WT1 Isoform with Antagonistic Effects against Major WT1 Isoforms
Source: PLoS One. 2015 Jun 19;10(6):e0130578. doi: 10.1371/journal.pone.0130578 (PMC4474557; doi:10.1371/journal.pone.0130578)

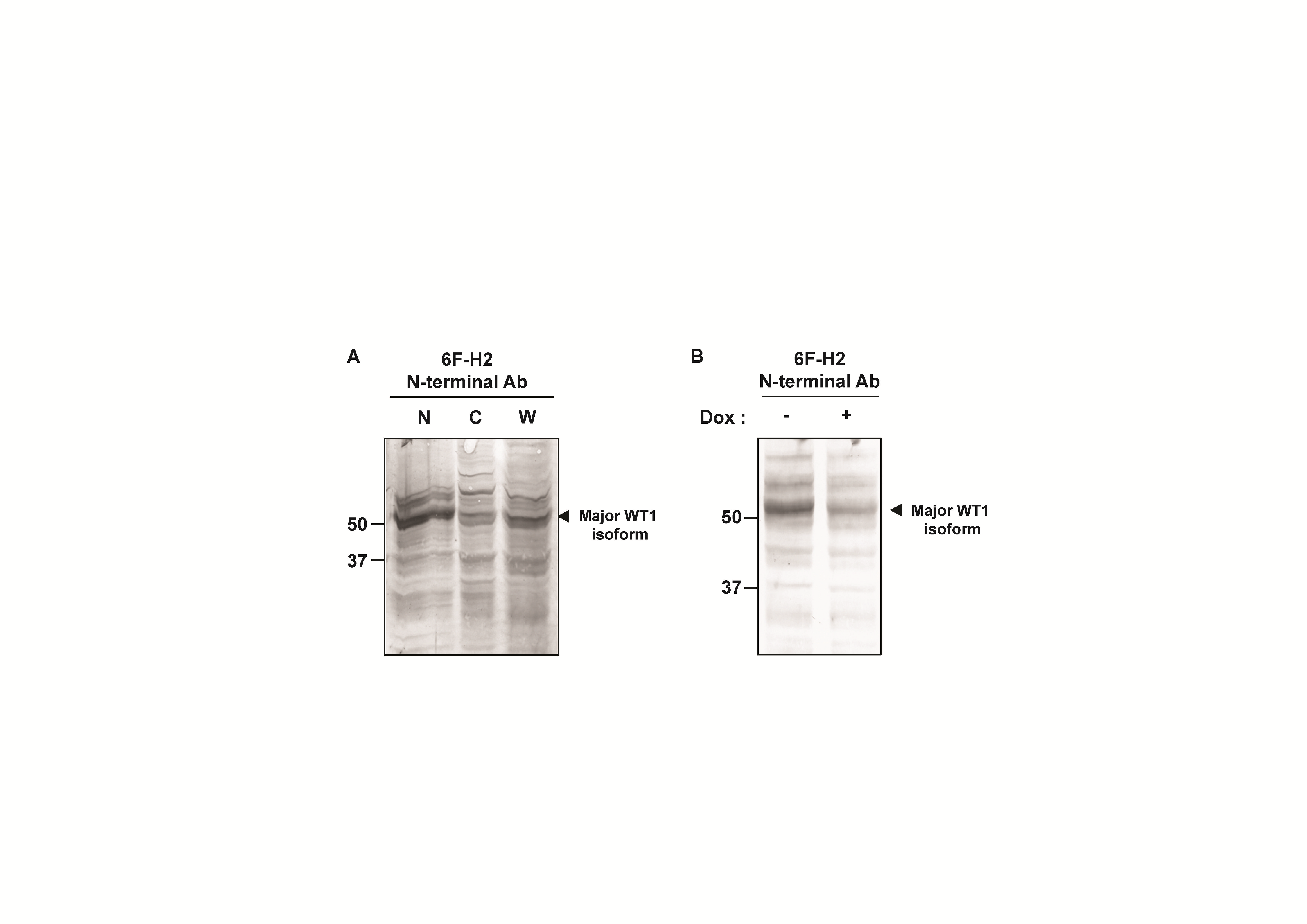

Supplement: S1 Fig — (A) Nuclear and cytoplasmic fractions of K562 cells were isolated and then examined for WT1 protein expression by Western blot analysis with 6F-H2 (specific for the N-terminal region of WT1 protein) antibody. N and C indicate the nuclear and cytoplasmic fractions of K562 cells, respectively. W indicates whole cell lysate from K562 cells. (B) WT1 protein expression was examined in Dox-treated K562 cells, where Ex4a(+)WT1 mRNA increased. K562 cells were treated with 4 μM of Dox for 24 h and analyzed for WT1 protein expression by Western blot analysis with 6F-H2 antibody (Left). (A-B) Results are representative of three independent experiments. (TIF) [file pone.0130578.s001.tif]

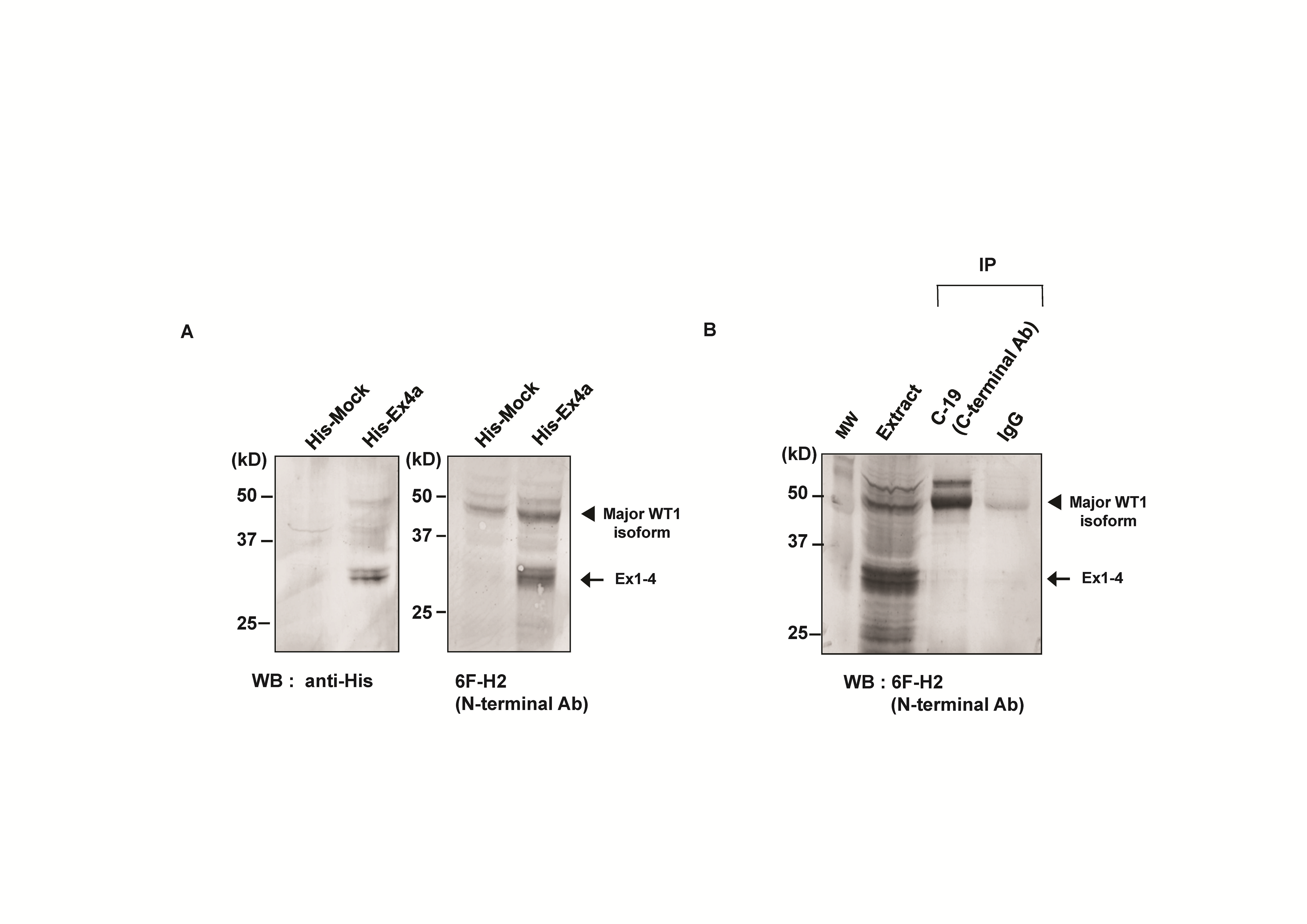

Supplement: S2 Fig — (A) Establishment of K562 cell clones transduced with His-tagged Ex4a(+)WT1 isoform. Expression of His-Ex4a(+)WT1 proteins were examined by Western blot analysis with anti-His tag (Left) or 6F-H2 (specific for the N-terminal region of WT1 protein) (Right) antibody. MW represents molecular weight marker. Arrowheads and arrows indicate major WT1 protein isoforms and 30-KDa His-tagged truncated Ex1-4 WT1 protein, respectively. (B) Immunoprecipitation assay. Cell lysates from K562-His-Ex4a(+)WT1 cells were subjected to immunoprecipitation with C-19 (specific for the C-terminal region of WT1 protein) antibody or control non-immune IgG (IgG). The resulting immunoprecipitated complexes were separated by SDS-PAGE and analyzed with 6F-H2 (specific for the N-terminal region of WT1 protein) antibody. MW represents molecular weight marker. Arrowheads and arrows indicate major WT1 protein isoforms and 30-KDa His-tagged truncated Ex1-4 WT1 protein, respectively. Cell lysates are immunoblotted as a control (Extract). Results are representative of three independent experiments. (TIF) [file pone.0130578.s002.tif]
